# An Explainable AI-Based Deep Learning Framework with Spatial and Multi-Head Attention for Bubble Detection in Tire Shearography Images: An Industrial Case Study

**DOI:** 10.3390/s26216864

**Published:** 2026-10-30

**Authors:** Radhwan A. A. Saleh, Mehmet Zeki Konyar, Humam AbuAlkebash, Farid Al-Areqi, Huseyin Metin Ertunc

**Affiliations:** 1 Faculty of Engineering and Architecture, Nişantaşı University, Istanbul 34485, Türkiye; radhwan.saleh@nisantasi.edu.tr; 2 Interdisciplinary Research Center for Construction and Building Materials, King Fahd University of Petroleum and Minerals (KFUPM), Dhahran 31261, Saudi Arabia; 3 Department of Software Engineering, Kocaeli University, Kocaeli 41001, Türkiye; 4 DigiNova Informat Tech Res Inc., KOU Teknopark, Kocaeli 41275, Türkiye; 5 Robotics and Artificial Intelligence Program, Department of Electronics and Automation, Ford Otosan İhsaniye Automotive High Vocational School (FOIMYO), Kocaeli University, Kocaeli 41650, Türkiye; 6 Department of Biomedical Engineering, Kocaeli University, Kocaeli 41001, Türkiye; 7 Department of Mechatronic Engineering, Kocaeli University, Kocaeli 41001, Türkiye

**Keywords:** tire defect detection, attention modules, Grad-CAM, shap values, shearography, bubble defect detection, XAI

## Abstract

The safety and reliability of automobile tires depend directly on the early detection of internal defects, since undetected air bubbles between rubber layers can propagate into catastrophic in-service failures. Although digital shearography has become the preferred non-destructive testing technique for revealing such subsurface anomalies, its industrial interpretation still relies on manual expert assessment, which is slow, operator-dependent, and difficult to audit. This study proposes an explainable deep learning framework for automated bubble detection in tire shearography images. The framework couples a DenseNet201 backbone with two complementary attention modules: a Spatial Aggregation Pyramid (SAP) that refines multi-scale feature representations through dilated depth-wise separable convolutions, and a Multi-Head Attention (MHA) module that models long-range contextual dependencies, so that both fine-grained texture variations and global spatial cues are captured. The model was developed on a class-balanced set of 1162 shearography images (581 per class) from 174 tire treads at the Pirelli İzmit factory, obtained by randomly excluding part of the non-defective images. Under 5-fold cross-validation, the proposed DenseNet201 SAP MHA model reached a mean accuracy of 0.962 with a standard deviation of 0.006 and an AUC (area under the curve) of 0.9794, consistently outperforming the DenseNet201 and DenseNet201 SAP baselines across accuracy, precision, recall, and F1-score. The improvements were consistent across all five folds and were supported by statistical testing. To address the black-box concern inherent in safety-critical inspection, Gradient-weighted Class Activation Mapping (Grad-CAM) and SHapley Additive exPlanations (SHAP) were integrated, demonstrating that the model bases its decisions on diagnostically relevant regions rather than background artefacts. The framework therefore delivers not only high bubble detection accuracy but also a transparent and auditable diagnostic tool suitable for industrial quality control.

## 1. Introduction

The safety and reliability of tires are crucial to automobile performance and driving safety. Hidden defects in tires, such as bubbles, may result in catastrophic failures, endangering drivers and passengers. Furthermore, they can result in severe consequences, such as accidents causing harm or death. The mere presence of bubbles or other defects in the structure of tires compromises their reliability and performance. Bubble detection in automobile tires is thus a critical quality control procedure in tire manufacturing [1]. Identifying these defects during the manufacturing process is essential for avoiding accidents and ensuring public safety. Enhanced detection methods not only save lives but also increase consumer confidence in tire manufacturers, resulting in a safer driving experience [2].

From an economic standpoint, efficient and accurate detection of tire defects can significantly reduce expenses related to product recalls, warranty claims, and liability concerns. Early detection of defects allows manufacturers to handle quality issues during production, reducing waste and rework. As a result, deploying robust defect detection systems can result in significant cost savings and increased overall profitability for tire manufacturers. Furthermore, maintaining high-quality standards helps manufacturers retain their brand reputation and market competitiveness [3].

**Figure 1:**
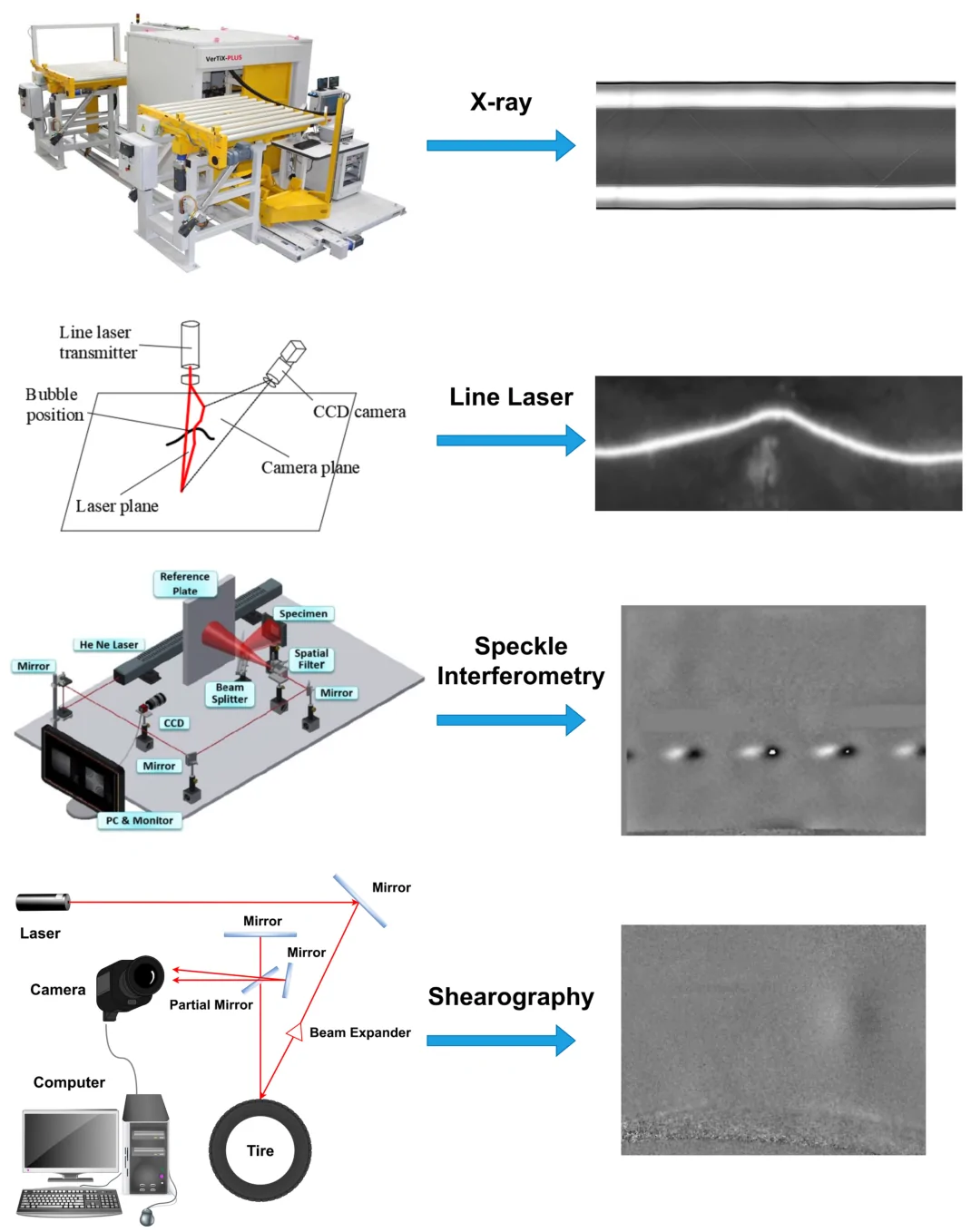
NDT techniques used for tire inspection and their corresponding image output.

Traditionally, several non-destructive testing (NDT) techniques were used to inspect the inner structure of tires, with notable examples including X-ray imaging, Speckle Interferometry, and digital shearography as shown in Figure 1. X-ray imaging, for instance, offers high-resolution images of a tire’s interior structure, exposing defects that are not visible externally [4]. However, it is costly, involves substantial radiation exposure, and requires extensive safety procedures, making it unsuitable for routine examinations [5]. Furthermore, X-ray imaging is more accurate in detecting other tire faults, such as foreign materials and misaligned layers [6]. Speckle Interferometry, another NDT technique, detects irregularities within tires using a laser beam and a charge-coupled device (CCD) camera, but the material’s density and geometry can restrict its effectiveness, resulting in inconsistent results. In contrast, Shearography uses laser interferometry to measure the deformation of the surface of a tire under stress, which can reveal the presence of interior defects. This method is extremely sensitive and can detect subtle irregularities that other techniques may fail to recognize [7]. In recent years, shearography has become known as a superior non-destructive testing technique. However, previous shearography image analysis relied on manual examination by qualified experts, which is time-consuming and prone to human error [8]. Figure 1 illustrates the NDT techniques used for tire inspection.

Shearography is frequently used in the rubber industry for quality control of tires belonging to sensitive vehicles such as racing cars and airplanes. The main focus of the laser shearography technique is the detection of air bubbles between the rubber layers. When hot rubber fabric layers are added on top of each other during tire production, if not enough care is taken, air bubbles will occur between the layers. Air bubbles between the layers may appear on the tread or sidewalls of the tire. Detection of air bubbles is done after the tire is baked and before the storage process. Since waves caused by shaking in hot tires can be perceived as bubbles, for correct control, the tire coming out of the baking oven must be cooled to ambient temperature and entered into the control machine without any impact. CCDs and complementary metal-oxide semiconductors (CMOS) are frequently used in shearography systems to record photographs of the tire’s surface while testing. These sensors are critical for turning reflected laser light into high-resolution digital images, which are subsequently evaluated for any defects. The procedure starts with providing stress to the tire, either through mechanical pressure, thermal stimuli, or vacuum loading, which causes minor deformations in its surface. As the tire deforms, the laser beam projected onto its surface generates interference patterns that are detected by the CCD or CMOS sensor. The generated images, called shearograms, show the strain distribution on the tire’s surface. These strain patterns reveal differences that may suggest internal flaws, such as bubbles or spots within the tire layers. After obtaining 8 different images taken from 45-degree angles, the quality control operator performs bubble control according to his experience. The quality control process is more or less effective depending on the experience and working hours of the operator. To overcome this issue, conventional image processing techniques were first used to improve and analyze shearography images. Edge detection, filtering, and thresholding were employed to identify potential defects in the tire structure. While these strategies increased defect visibility, they frequently lacked the sophistication required to reliably distinguish real defects from benign anomalies [9].

As the technology developed, traditional machine learning (ML) methods gained popularity in defect detection. Support vector machines (SVMs), decision trees, and k-nearest neighbors (k-NN) were used to classify defects using features extracted from shearography images. These algorithms outperformed basic image processing in terms of accuracy, but they still required substantial feature engineering and were constrained by their inability to learn from raw data [10]. The advent of deep learning marked a major step forward in defect detection capabilities. Convolutional neural networks (CNNs), in particular, revolutionized the field by allowing for the automated extraction of significant information from shearography images. Deep learning models may learn complex patterns and representations from immense datasets, increasing the accuracy and robustness of defect detection systems. For instance, Mignot et al. [11] proposed a two-stage multimodal deep learning system that first localizes tire surface defects, including air bubbles, through instance segmentation and then classifies their severity, illustrating the expanding role of deep architectures in automated tire quality control.

Recent advancements in deep learning, especially the introduction of transformers and vision transformers (ViTs), have surpassed the performance of CNNs in various computer vision tasks [12]. ViTs leverage self-attention mechanisms to capture global context and relationships within the data, providing superior accuracy and robustness in image analysis. Despite the fact that these models outperform classic machine learning approaches or manual detection methods, there is still a pressing demand for more precise and reliable models. In parallel, the coupling of attention mechanisms with multi-scale feature fusion has become central to modern defect detection. The aggregated multi-level feature interaction fusion network (AMFNet) [13], for example, integrates channel and spatial attention with multi-scale features to detect defects of widely varying sizes on steel surfaces, confirming that the joint use of attention and multi-scale processing yields substantial accuracy gains. Likewise, F2MCANet [14] combines multi-channel attention with frequency-domain fusion to strengthen surface defect recognition, further demonstrating the adaptability of attention-based designs to fine-textured anomalies.

Another important concern for critical applications, such as bubble defect detection, which can result in serious safety risks and major loss of life, is the reliability of the detection system. Given that most bubble detection techniques in the literature operate as “black boxes” with no transparency into their decision-making processes, the necessity for a reliable and explainable artificial intelligence (XAI) model becomes essential [15]. The implementation of XAI approaches, such as saliency maps, SHapley Additive exPlanations (SHAP) values, and Gradient-weighted Class Activation Mapping (Grad-CAM), can highlight image regions that contribute the most to the model’s predictions, providing a visual explanation of how the model detects defects [16]. This transparency is required not only to improve model performance, but also to earn the trust of the manufacturers, engineers, and quality control individuals who rely on these systems [3]. Reinforcing this need, Ahangar et al. [17] applied Grad-CAM and SHAP across several manufacturing inspection tasks and showed that explainability aligns model decisions with physically meaningful defect mechanisms while supporting compliance with emerging regulations such as the EU AI Act. In a related effort, an explainable hybrid framework paired a fusion-based detector with Grad-CAM to deliver both high detection accuracy and transparent localization of steel surface defects [18], echoing the dual objective of performance and interpretability pursued in this work. In addition to enhancing defect detection, the utilization of XAI in tire inspection can help with regulatory compliance. Regulatory agencies frequently request detailed documentation and justification for methods used in safety-critical inspections. Manufacturers can use XAI to offer thorough information on how specific defects are identified, assuring transparency and compliance with industry standards. This transparency not only helps with regulatory compliance but also boosts confidence in automated inspection systems among stakeholders [19].

**Figure 2:**
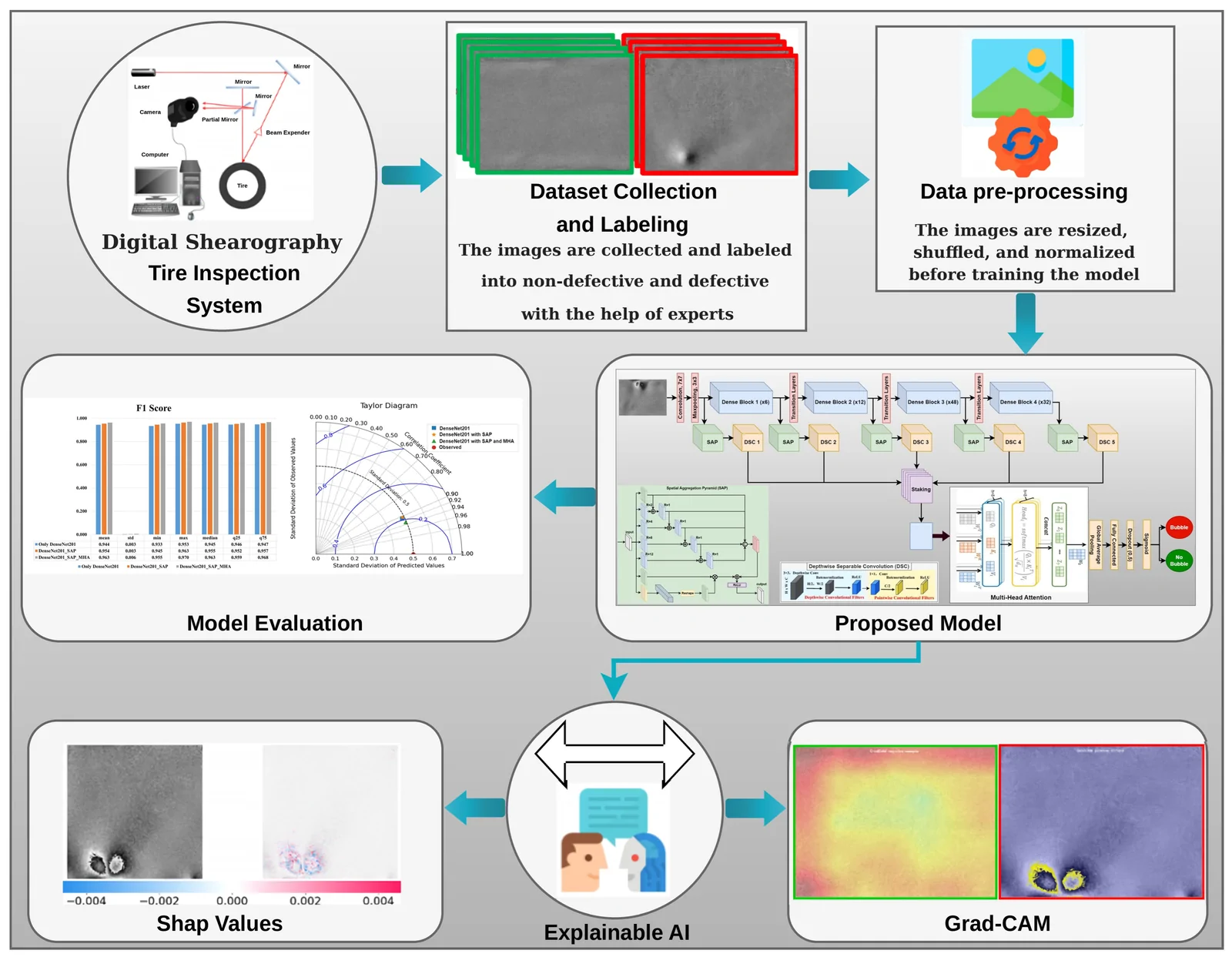
The proposed framework structure.

Inspired by the defined superiority of shearography over other NDT methods, the urgent need for accurate and reliable bubble detection systems, and the lack of explainable AI (XAI) models in this domain at the moment, this work focuses on creating cutting-edge solutions that improve defect detection processes’ transparency and accuracy. This study investigates the integration of a spatial aggregation pyramid (SAP), multi-head attention mechanism (MHA) and XAI approaches to detect bubbles in tire shearography images. By leveraging the capabilities of both techniques, we aim to highlight how these technologies can overcome the constraints of traditional inspection methods to offer a robust, more efficient, accurate, and interpretable solution for automated tire inspection. The proposed approach of this study combines advanced convolutional network techniques with attention mechanisms to improve feature extraction and the model’s ability to detect minuscule variations in image textures associated with tire bubbles. The DenseNet201 architecture is selected as a backbone of the proposed structure for its ability to propagate and reuse features rapidly, which is critical for capturing complex patterns in shearography images. In addition, DenseNet201 achieved the best performance among six transfer learning backbones on the same dataset in our previous study [20]. This choice is further motivated by recent evidence that DenseNet-based backbones, when augmented with attention modules, alleviate the suppression of small targets that commonly occurs in deep networks and thereby improve the detection of subtle defects [21]. Feature maps are generated at five separate levels from DenseNet201 blocks, ranging from initial convolutional layers to advanced dense blocks. Two attention modules are deployed to further enhance these features: the MHA mechanism and the SAP module. This enables the model to capture both local and global contextual information for more accurate defect recognition, as well as to focus on crucial locations. Figure 2 depicts a graphical depiction of the proposed approach workflow. A detailed description of the model workflow is provided in Section 3.2.

The contributions of this research article can be briefly summarized as follows:

- We adapt a SAP module from existing multi-scale dilated convolution designs, which refines feature representation through multi-scale dilated depth-wise separable convolutions, enabling the network to capture bubble signatures across a wide range of spatial scales at low computational cost.
- We combine SAP and MHA with a DenseNet201 backbone for bubble classification in tire shearography images and evaluate the incremental contributions of the modules through an ablation study across three model variants.
- The differences between the model variants are examined with statistical tests, and the results are interpreted with the small number of cross-validation folds in mind.
- We integrate post-hoc explainability (Grad-CAM and SHAP) into shearography-based tire bubble detection, which, to the best of our knowledge, has not been reported before. By combining Grad-CAM and SHAP, we provide both spatial (region-level) and feature-level evidence that the model attends to genuine defect cues, addressing the transparency and regulatory-traceability requirements of safety-critical inspection.
- We build on the real industrial tire shearography dataset introduced in our previous work [20], comprising images of 174 tire treads spanning roughly fifty design patterns from the Pirelli İzmit factory, and we describe in detail the acquisition protocol that removes thermally induced pseudo-defects and the class-balancing procedure (1162 images), improving label reliability and the transparency of the experimental setup.
- The framework is delivered as an automated end-to-end system that has been deployed on the shearography inspection line at the Pirelli İzmit factory, and its computational cost, including the explainability overhead, is reported.

The following sections of this study will look at the specific approaches used, the performance of the suggested approach, and its implications for tire manufacturing.

## 2. Literature Review

The identification of tire defects is crucial for both human safety and vehicle performance. Advances in imaging techniques and defect detection technologies have greatly improved the ability to detect tire anomalies such as bubbles, foreign materials, and structural inconsistencies. This literature review will delve into studies that attempted to detect tire defects, especially bubbles, and explore the findings and effectiveness of each imaging technique, as well as the implementation of various detection algorithms ranging from traditional image processing to advanced machine and deep learning algorithms. The review highlights and provides a comprehensive understanding of current tire defect detection technologies.

### 2.1. X-Ray-Based Imaging

X-ray imaging has been commonly employed to detect tire defects. Zhang et al. [22] present an approach that combines total variation and edge detection techniques. The authors use total variation to reduce noise and retain edges in X-ray images, subsequently employing edge detection techniques to discover potential defects. An additional investigation by the same authors [23] examined the application of wavelet multiscale edge detection to evaluate faulty edges in tires, providing a framework for effectively distinguishing defect regions from the background. Zheng et al. [24] proposed a technique that uses an enhanced extremum filter and locally adaptive-threshold binaryzation to detect clear and blur impurities, as well as clear bubble defects in tire X-ray images, whereas blur bubbles were difficult to distinguish from the background. Another research effort by Zhu and Ai [25] develops a deep learning-based technique for automatically detecting tire defects in X-ray images. The approach leverages a deep CNN to learn and recognize features associated with faults, resulting in excellent defect identification accuracy. However, the proposed strategy necessitates extensive computer resources and an immense labeled dataset. Wu et al. [26] presented a novel tire defect detection approach that uses Faster R-CNN to detect and localize defects in tire X-ray images. The discovered approach only detects bubbles in the sidewall, not in the tread itself. We previously [3] investigated tire defect detection in X-ray images using machine learning methods. Although we obtained encouraging results with a few-labeled dataset, the bubble defect type was not included. Since X-ray images show density differences in the tire’s internal structure, whereas shearography images show the surface strain distribution under vacuum loading, the two modalities use different inspection machines, target different defect types and produce images of entirely different appearance; therefore, models developed for X-ray images are not directly applicable to shearography. These investigations show that X-ray imaging is effective in detecting various tire defects, but the primary drawback is the radiation hazard associated with these imaging systems, which necessitates the installation of additional protective equipment. Furthermore, the heterogeneity of tire materials and structures limits the identification of bubbles in radiography images.

### 2.2. Laser-Based Imaging

Several laser-based imaging techniques have recently been established and utilized to detect tire defects, particularly bubbles. Wang et al. [27] developed a quantitative post-processing module for online automatic image inspection, combining image processing with automated inspection machines to standardize defect detection, however, it had potential challenges with certain sidewall types. Yang et al. [28] suggested a method that combined line laser scanning and machine vision to detect surface bubble defects by evaluating tire surface profiles, however, it was only effective for surface defects and required laser calibration. Chang and Wang [9] used CNN and Faster R-CNN for defect recognition, employing deep learning to improve accuracy and manage a wide range of defect types and sizes, though requiring large labeled datasets and computational resources. Yang et al. [29] used speckle interferometry with coherent light to detect tire surface deformations, proposing an improved TY-FPN network to deal with low contrast and small object size challenges; however, it was susceptible to external factors such as vibrations and temperature fluctuations. Chang et al. [30] also investigated incremental learning with YOLO for detecting defects in speckle images, which adjusts over time to new data but may initially be less accurate. Furthermore, Chang et al. [8] presented a quality assessment approach for shearography images that uses ensemble hybrid Faster R-CNN models to improve robustness and accuracy, but at a significant computational cost. Finally, Zheng et al. [31] used 3D laser scanning technology to acquire and analyze the tire surface and structure in three dimensions, which improved detection accuracy while requiring advanced equipment and processing massive data volumes eventually affecting the speed.

More recently, Wu et al. [32] employed a U-Net network to automatically segment and measure defect size in shearography images of composite materials, achieving high-precision sizing for a task that previously depended on manual inspection. Such shearography deep learning studies, however, remain almost exclusively focused on composite structures, leaving tire inspection largely unaddressed. Similarly, Li et al. [33] designed a spatiotemporal object detection network for near-surface defect detection in composites, trained solely on simulated shearography phase-map sequences to overcome the high cost of acquiring real datasets. This reliance on simulated or composite-material data underscores the persistent absence of publicly available, annotated shearography datasets for tires, a gap to which the present study contributes.

### 2.3. Detection Method-Based

The advancement of tire defect detection methods ranges from traditional image processing to advanced attention-based approaches. Initially, Zhang et al. [22] used total variation decomposition, curvelet transform-based image enhancement, and Canny edge detection on radiography images to detect foreign objects and bubble defects with higher accuracy than conventional Sobel and Laplacian of Gaussian (LoG) operators. Another study [23] using wavelet multiscale analysis obtained excellent detection rates and precise localization across 400 selected images from a dataset of 3700, demonstrating its effectiveness for multi-textural radiography images. Yung-Cheng Wang and colleagues [27] developed a quantitative post-processing module that was implemented into an online automatic inspection system, improving inner bubble defect detection with an accuracy of 85% over 5000 images. By efficiently isolating defects from the background, an improved extremum filter and locally adaptive-threshold binarization approach showed 97% precision and 95.7% recall on 280 tire X-ray images [24]. Another approach [28] used machine vision and line lasers to obtain a 93% detection accuracy by studying the geometric properties of bubble defects in tire surfaces. In our earlier work, Saleh et al. [34] introduced a dataset of tire shearography images collected at the Pirelli İzmit factory and used radiomics features with classical machine learning classifiers to detect air bubbles. On a 1000-image dataset, a multilayer perceptron (MLP) achieved an accuracy of 85.90%, showing that machine learning methods can identify tire bubbles in shearography images with minimal training data. Subsequently, in our another work, Saleh et al. [20] compared six transfer learning backbones on an extended version of this dataset, with DenseNet201 achieving the best results, exceeding 95% accuracy.

A machine learning-based model involving the Gray Level Co-occurrence Matrix (GLCM) for feature extraction was evaluated on 1511 labeled images and achieved 99.34% accuracy [35]. Deep learning algorithms improve detection capabilities by automatically learning from preprocessed samples and achieving excellent recognition rates [25]. Using CNNs and Faster R-CNN, researchers in [8] and [9] achieved detection accuracy of 86.87% and 89.16% for sidewall and tread bubbles, respectively. The use of hybrid models combining CNNs and Faster R-CNN for tire shearography images improved accuracy while decreasing false positives. A faster R-CNN-based technique achieved a 95.4% recognition rate by preprocessing images and incorporating convolutional features [26]. An enhanced Faster R-CNN-FPN framework showed increased detection precision for small objects in tire crowns, with mAP gains of 2.08% [29]. Incremental learning with the YOLO architecture [30] obtained a sensitivity of 98.56% by iteratively enhancing detection capabilities semi-automatically. Finally, a novel 3D laser scanning technique based on deformable attention mechanisms demonstrated significant advances in precision, with mAP scores of 0.356, 0.409, and 0.691, highlighting the ongoing evolution and improvement of detection approaches in tire production [31].

These studies, while producing remarkable outcomes, often come with trade-offs concerning detection speed and computing cost. Some focused on accuracy, or recall, but rarely all metrics. Some studies utilized object detection algorithms that, while effective, were slow and required considerable processing power. This is less efficient when the main goal is to classify whether a tire contains a bubble or a fault or not. Our research aims to solve these limitations by implementing a state-of-the-art multi-scale attention model that offers improved detection efficiency and accuracy, resulting in a more balanced and robust solution for tire defect detection.

## 3. Methodology and Materials

A thorough explanation of the proposed methodology is given in this section. The preprocessing techniques and dataset description are presented first. The suggested structure and its essential components are covered in the subsection that follows.

### 3.1. Dataset and Preprocessing

**Figure 3:**
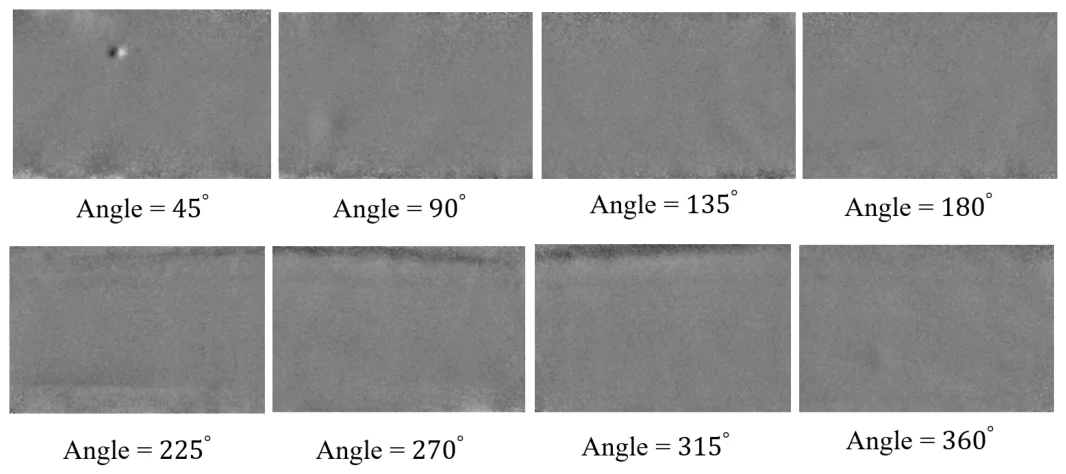
Shearography images for one tire in different angles.

The Pirelli Automobile Tires facility in Izmit, Kocaeli, Türkiye provided the dataset for this study, which was collected and labeled with expert assistance. The dataset consists of shearography images of 174 tire treads. For every tire tread, eight images are captured, each of which involves rotating the tire 45 degrees. A sample of the eight images for a single tire is shown in Figure 3. Each tire image was labeled independently because defects like bubbles are often localized to specific sections. For example, only one of the eight images in Figure 3 contains a bubble, while the remaining seven are defect-free. Consequently, Data splitting was performed at the image level, so images of the same tire may appear in different folds. Since the eight images cover non-overlapping 45° sectors, no physical region of a tire appears in more than one image; however, images of the same tire share the tread pattern and acquisition conditions, which may make the cross-validation estimates optimistic.

**Figure 4:**
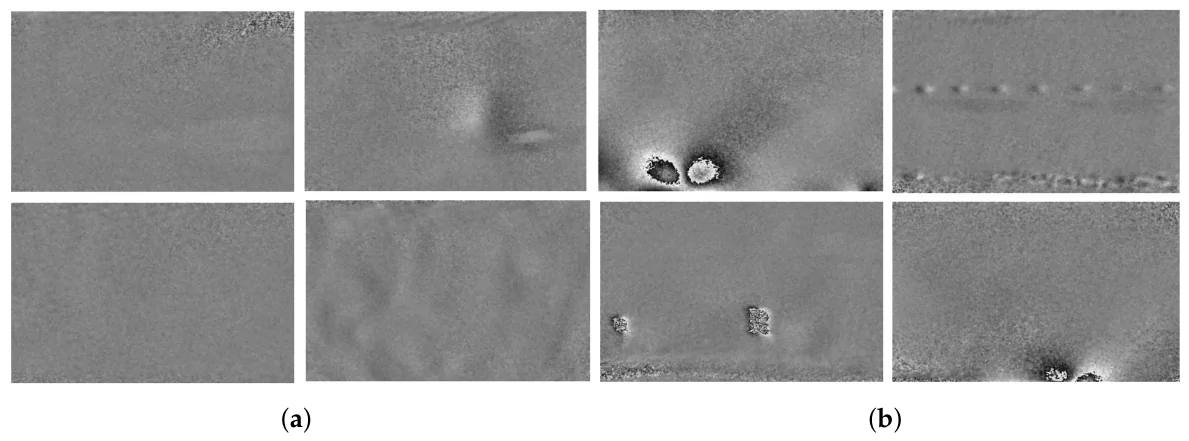
Samples of shearography images for non-defective and defective tires classes. (**a**) Shearography images for non-defective tire class; (**b**) Shearography images for defective tire class.

High tire rubber temperatures and mechanical vibrations during production line crossings can cause temporary surface fluctuations that resemble air bubbles. While quality controllers re-scan these tires after they cool, the raw system logs still record the initial false-positive images. To prevent the AI model from learning spurious features, these transient artifact images were eliminated during dataset preparation. The same procedure is followed when the system is used on the production line: when an acquisition shows such transient artifact patterns, the operator disregards the model’s prediction, regardless of whether it is correct, and the tire is sent back for cooling and re-imaging, so that the final decision is always based on an artifact-free acquisition. Following this filtering step, the dataset contained 581 defective images and 811 non-defective images. To establish a balanced class distribution, 581 images were selected randomly from the non-defective class to match the defective count. Balancing was applied to prevent the classifier from being biased toward the majority class during training and to keep accuracy directly interpretable; since the original imbalance is moderate (about 1.4:1) and the non-defective images were selected randomly, the variety of tread patterns and acquisition conditions in this class is preserved. The expected performance under the original class distribution is reported in the results. Representative shearography samples for both classes are shown in Figure 4. Finally, as standard in the literature [36], all images were preprocessed via normalization and resizing to fit the model’s input dimensions.

### 3.2. Proposed Model

**Figure 5:**
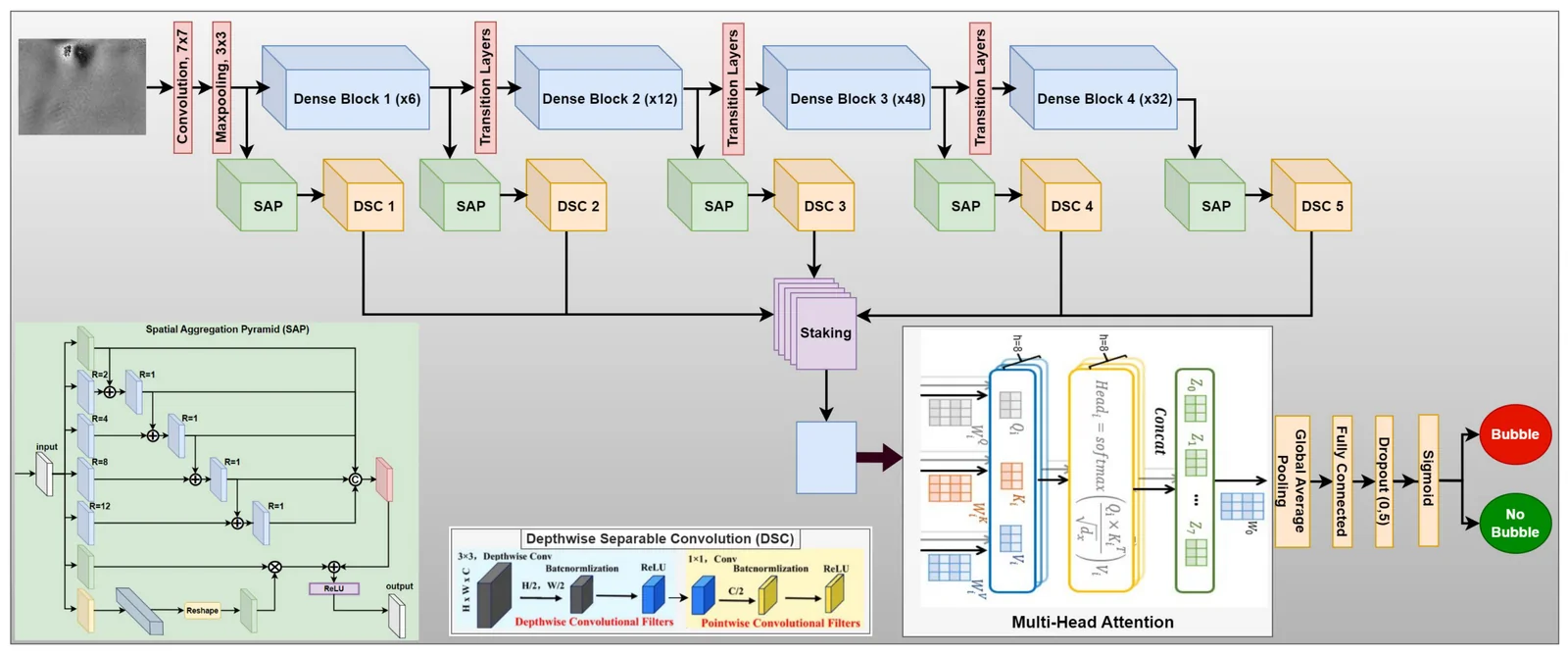
Proposed Model Structure.

The proposed architecture meant to detect bubbles in this study makes use of a variety of advanced convolutional network approaches to improve feature extraction and the model’s capacity to detect tiny variations in image textures associated with bubbles. It relies on the DenseNet201 architecture as its backbone due to its rapid feature propagation and reuse, which is critical for collecting complex patterns in images. DenseNet201 is incorporated without the top classification layers, allowing the model to learn detailed feature representations at various sizes. The proposed model’s backbone generates feature maps at five unique levels, each representing outputs from different blocks of the DenseNet201 architecture: the initial convolutional layers, the second, third, and fourth dense blocks, and the final dense block. Each feature map is then processed using a specific attention-based method that improves the detection of relevant characteristics while suppressing less important data. This selective attention processing is critical for accurately detecting and recognizing bubbles in imaging data. Thereafter, the proposed attention modules’ outputs are aggregated and subjected to global average pooling to reduce dimensionality while maintaining the most important information. This pooled feature vector is fed into a dense layer of 512 units, followed by a dropout layer to prevent overfitting. Finally, the network outputs the likelihood of bubble occurrence via a sigmoid activation function, making it perfect for binary classification applications. Figure 5 shows the detailed architecture of this proposed model.

### 3.3. Spatial Aggregation Pyramid (SAP)

Deep learning demands useful feature extraction modules across several scales for tasks that demand fine-grained recognition, such as image bubble detection. To solve this challenge, the proposed model includes a Spatial Aggregation Pyramid (SAP) module. The SAP module improves the model’s performance by refining feature representation using multi-scale processing. The SAP module employs depth-wise separable convolutions to efficiently extract multi-scale features at varying dilation rates. This method enables the sophisticated capture of both small and large details inside the image, which improves the semantic representation of feature maps. By including an attention-refining mechanism, the module selectively draws attention to more relevant characteristics while reducing less important features. This advanced mix of multi-scale feature extraction and specialized attention considerably improves the model’s capacity to detect and classify complicated patterns, such as bubbles in various imaging contexts.

While multi-scale dilated convolution modules such as Atrous Spatial Pyramid Pooling (ASPP) [37], and DenseASPP [38] have established the paradigm of capturing multi-scale context through dilated convolutions, the SAP module proposed here differs in three specific ways. First, SAP employs depth-wise separable dilated convolutions rather than standard dilated convolutions, reducing computational cost while preserving multi-scale representation capability. Second, SAP stacks these convolutions in a cascaded manner with increasing dilation rates and residual pixel-wise additions between stages, in contrast to the parallel branches of ASPP and the dense connections of DenseASPP; this cascaded design enables progressive feature refinement across scales. Third, SAP incorporates a dedicated attention refinement branch that uses global average pooling to generate channel-wise attention weights, a component absent from ASPP and DenseASPP, which rely solely on multi-scale aggregation without explicit attention modulation. These design choices were empirically tuned for bubble detection in tire shearography images, a task with low-contrast, fine-grained defects that differs fundamentally from the natural scene segmentation tasks for which ASPP and DenseASPP were developed.

The SAP module starts the process by compressing high-level input features $X\in R^{H\times W\times C}$ with a 1×1 convolution. This first step is critical for lowering dimensionality while maintaining important information.

(1) $$Z_{1}=\delta \left(\mathrm{BN}\left(\mathrm{Conv}\left(X,W_{1}\right)\right)\right),$$

where Conv(·,·) denotes the convolution operation, BN represents batch normalization, δ is the ReLU activation function, and $W_{1}$ are the learnable parameters of the convolution filter.

Following that, the module performs multi-scale feature extraction using depth-wise separable convolutions with dilation rates increasing from 2 to 12. These convolutions are intended to capture a wide variety of spatial information while requiring minimal processing resources.

(2) $$Z_{i}=\delta \left(\mathrm{BN}\left(\mathrm{SepConv}\left(Z_{i-1},W_{i},r_{i}\right)\right)\right)⊕Z_{i-1},$$

where:

- $\mathrm{SepConv}(Z_{i-1},W_{i},r_{i})$ represents the depthwise separable convolution operation applied to the feature map $Z_{i-1}$ with learnable weights $W_{i}$ and dilation rate $r_{i}$.
- $r_{i}$ specifies the dilation rate, which adjusts the spacing between kernel elements, enabling the capture of information from increasingly larger receptive fields without a proportional increase in computational complexity.
- δ denotes the ReLU activation function, and BN stands for batch normalization, which normalizes the output of the convolution for better stability and performance of the network.
- ⊕ signifies pixel-wise addition, which effectively integrates the extracted features with those processed in previous steps, enhancing the multi-scale feature representation within the module.

The retrieved features from different scales are then aggregated via pixel-wise addition, combining information from many contexts into a cohesive feature map. This integration is critical for ensuring a complete representation of both local and global features. An attention refinement branch has been added to improve the semantic representation even more. This branch employs global context information to fine-tune the feature map, ensuring that the model concentrates on the most informative regions of the image. This process is mathematically described as follows:

(3) $$Z_{6}=\sigma \left(\mathrm{BN}\left(\mathrm{Conv}\left(\mathrm{GAP}\left(X\right),W_{6}\right)\right)\right)⊗X,$$

where:

- GAP(X) refers to the Global Average Pooling operation, which compresses the spatial dimensions of X by averaging over each channel, resulting in a feature vector that encapsulates global information across the spatial extent of the input.
- $\mathrm{Conv}(\cdot ,W_{6})$ is a convolution operation with learnable parameters $W_{6}$, which transforms the global feature vector into a context vector suitable for guiding the attention mechanism.
- σ represents the sigmoid activation function, which scales the output of the convolution into a [0, 1] range, enabling it to act as a gating mechanism in the attention process.
- ⊗ denotes pixel-wise multiplication, which modulates the original feature map X with the attention signals generated from the global context, selectively enhancing features based on the extracted global cues.

This attention mechanism selects features that are more relevant to the task at hand, hence enhancing the model’s accuracy and interpretability. The SAP module’s final output is formed by concatenating the multi-scale features. These concatenated features are then coupled with the enhanced attention feature map via pixel-wise addition, resulting in a robust and detailed feature representation that is suitable for subsequent classification tasks.

(4) $$\mathrm{Final}\;\mathrm{Output}=\left(\underset{i}{⨁}Z_{i}\right)⊕Z_{6},$$

where ⨁ denotes the concatenation operation.

**Figure 6:**
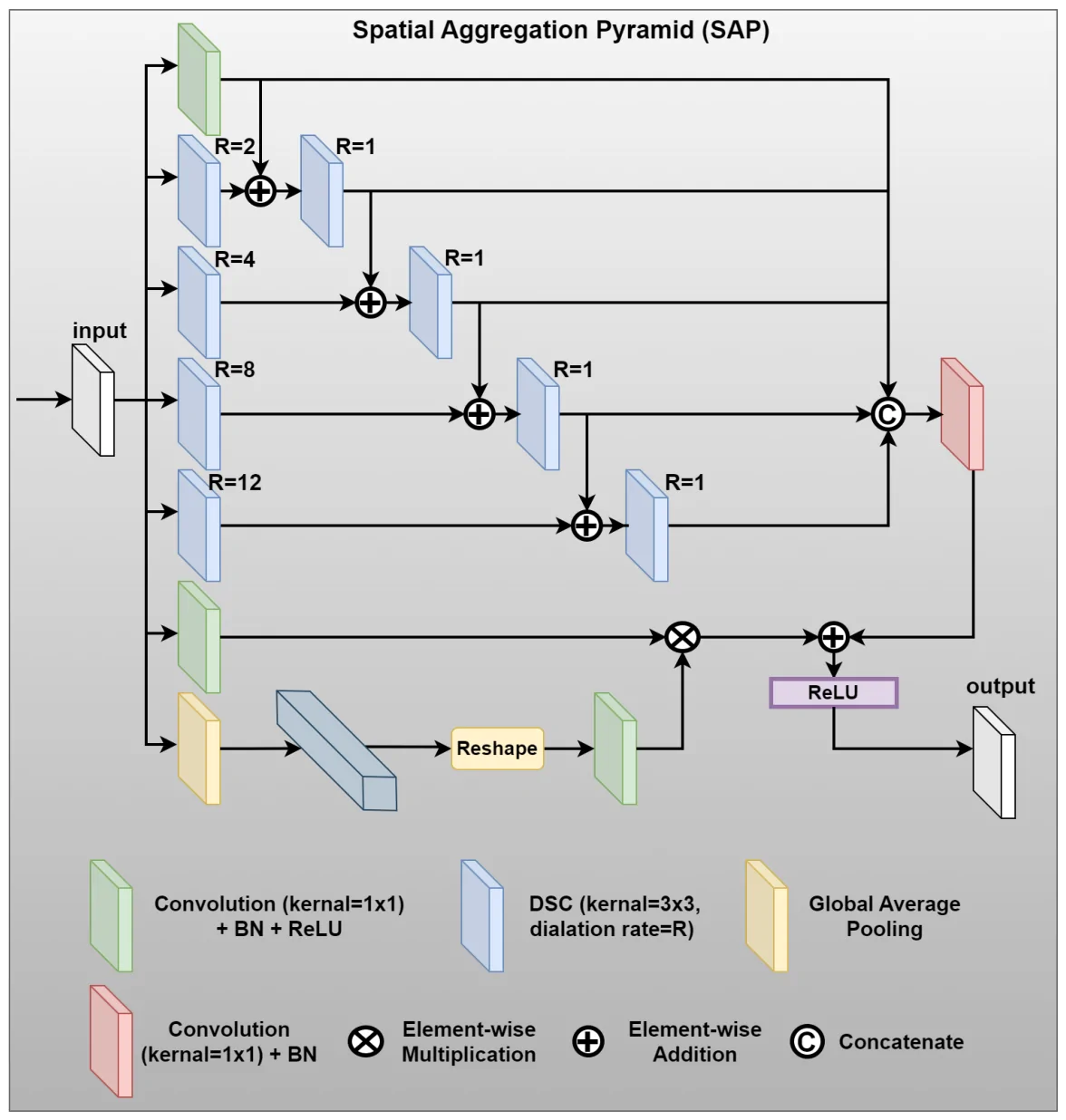
Spatial Aggregation Pyramid (SAP) Structure.

The visual representation of the SAP module, as provided in Figure 6, helps in understanding the flow of operations and the combination of different components to achieve a rich semantic feature map.

### 3.4. Multi-Head Attention Mechanism

The five SAP outputs are first aligned to a common spatial resolution of 7×7 with 256 channels by the depth-wise separable convolution (DSC) blocks as illustrated in Figure 5. DSC 1, which receives the feature map from the initial convolutional layers (56×56), applies a stride of (8,8); DSC 2, which receives the feature map from Dense Block 1 (28×28), applies a stride of (4,4); DSC 3, which receives the feature map from Dense Block 2 (14×14), applies a stride of (2,2); and DSC 4 and DSC 5, which receive the feature maps from Dense Block 3 and Dense Block 4 (7×7), respectively, apply no downsampling as they are already at the target resolution. The aligned maps are then stacked into a tensor of shape (batch,5,7,7,256), where the second dimension indexes the five scales, and multi-head attention is applied over this stacked representation with 8 heads and a key dimension of 64. The attention output is concatenated across heads and averaged across the five-scale axis to produce a single fused 7×7×256 feature map, which is subsequently passed to the classification head.

The multi-head attention mechanism is a critical component of the model, improving its capacity to properly analyze and integrate data from various feature maps. It accomplishes this by focusing on different aspects of the feature sets utilizing parallel attention heads [39]. The process is mathematically defined as follows:

(5) $$\mathrm{MultiHead}\left(Q,K,V\right)=\mathrm{Concat}\left({\mathrm{head}}_{1},\ldots ,{\mathrm{head}}_{h}\right)W^{O},$$

where each head (${\mathrm{head}}_{i}$) is computed as:

(6) $${\mathrm{head}}_{i}=\mathrm{Attention}\left(QW_{i}^{Q},KW_{i}^{K},VW_{i}^{V}\right),$$

and the Attention function is defined by the scaled dot-product attention:

(7) $$\mathrm{Attention}\left(Q,K,V\right)=\mathrm{softmax}\left(\frac{QK^{T}}{\sqrt{d_{k}}}\right)V.$$

In these equations:

- Q, K, and V represent the query, key, and value matrices respectively, which are derived from the feature maps being integrated.
- $W_{i}^{Q}$, $W_{i}^{K}$, and $W_{i}^{V}$ are the parameter matrices for the *i*-th attention head, transforming the input feature maps into different subspaces for queries, keys, and values.
- $W^{O}$ is the output transformation matrix that combines the outputs of all attention heads into a single feature map.
- $d_{k}$ is the dimensionality of the key vectors, and it is used to scale the dot products, aiding in training stability and performance.
- Concat(·) refers to the concatenation of the attention heads’ outputs, which is then transformed by $W^{O}$.

The utilization of multiple attention heads provides diverse representations of the input, which, when combined, contribute to a more comprehensive understanding of the data’s contextual relationships [40].

## 4. Experiments and Results

All of the studies were conducted on a PC running Windows 11 with 32 GB of RAM and an NVIDIA GeForce RTX 4060 8GB GPU. Furthermore, the pipeline for the experiments was created in the Tensorflow 2.12 environment. The input size was 224×224×3. Images were normalized by rescaling pixel values to [0,1]. The DenseNet201 backbone was initialized with ImageNet pretrained weights. Training used the Adam optimizer with a learning rate of $1\times 10^{-5}$, a batch size of 2, and 100 epochs, with binary cross-entropy loss and L2 regularization ($1\times 10^{-4}$) on the dense layers. A dropout rate of 0.5 was applied before the final classification layer. No data augmentation was applied; the only preprocessing steps were normalization and resizing. Evaluation used 5-fold cross-validation.

### 4.1. Evaluation Metrics

Evaluation metrics are crucial for evaluating the effectiveness of machine learning models. The confusion matrix, which classifies the model’s predictions into True Positives (TP), False Positives (FP), True Negatives (TN), and False Negatives (FN), is one of the key measures. Derived measures like accuracy, precision (positive predictive value, PPV), recall (true positive rate, TPR), and F1-score provide additional insights into how effective the model is. Furthermore, the capacity of a model to distinguish between classes is measured by the ROC curve (Receiver Operating Characteristic) and the corresponding AUC [41]. Although less frequently used, the Taylor diagram is helpful in complex evaluation circumstances as it offers a visual tool for comparing multiple models based on metrics such as correlation, standard deviation, and error [42].

(8) $$\begin{array}{c} Accuracy=\frac{TP+TN}{TP+TN+FP+FN} \end{array}$$

(9) $$\begin{array}{c} Precision\;\left(PPV\right)=\frac{TP}{TP+FP} \end{array}$$

(10) $$\begin{array}{c} Recall\;\left(TPR\right)=\frac{TP}{TP+FN} \end{array}$$

(11) $$\begin{array}{c} F1\_score=2\cdot \frac{Precision\cdot Recall}{Precision+Recall} \end{array}$$

### 4.2. Classification Results

**Figure 7:**
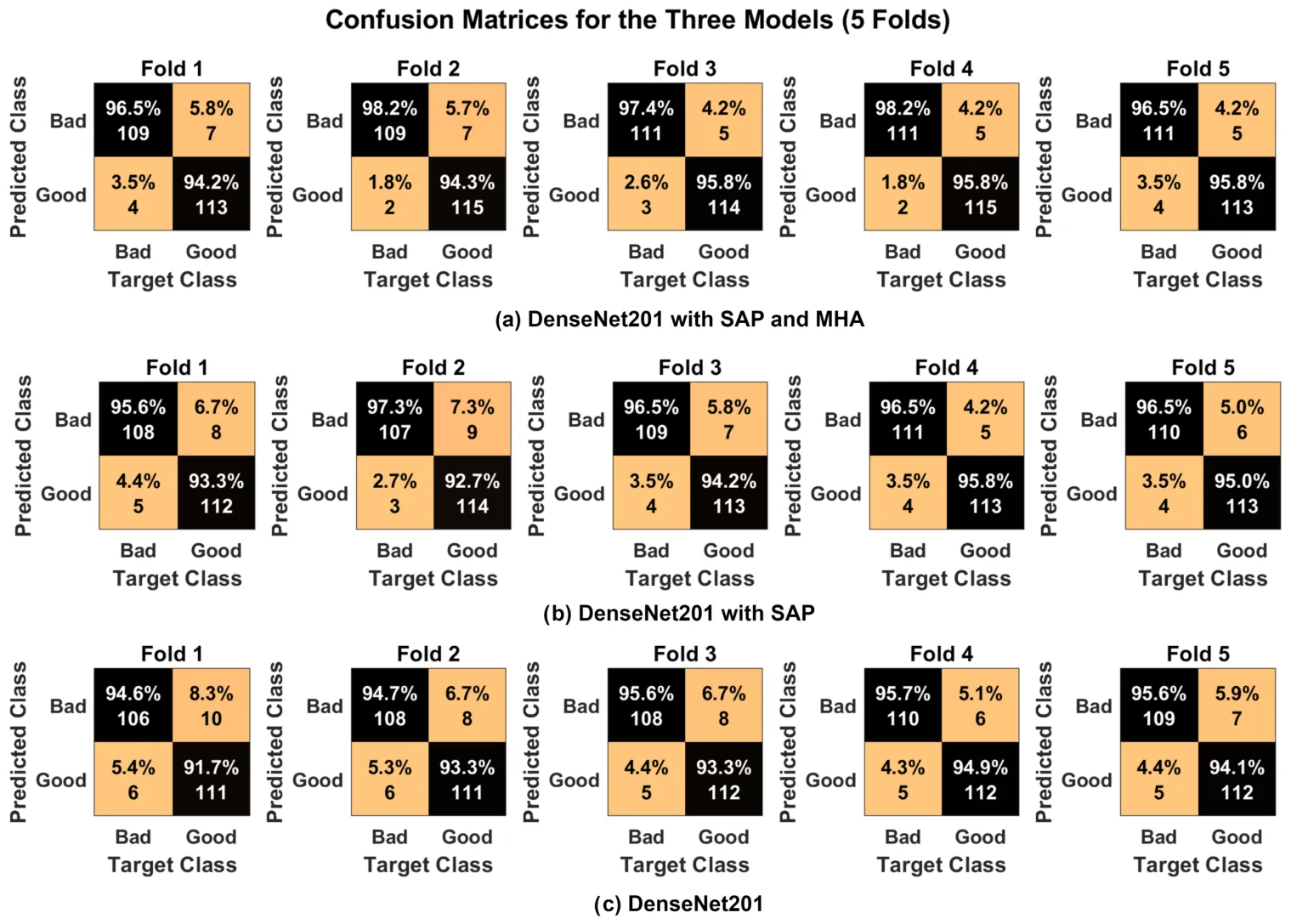
Confusion matrices for DenseNet201, DenseNet201_SAP, and DenseNet201_SAP_MHA on 5-fold cross-validation.

The performance of the proposed model, DenseNet201_SAP_MHA, was evaluated using 5-fold cross-validation. Furthermore, results were compared with those of two further model variations, DenseNet201 and DenseNet201_SAP, which were produced by deleting the SAP and MHA modules from the proposed DenseNet201_SAP_MHA model. This comparison demonstrates how these elements affect the model’s performance. The three variants reflect the stacking order of the modules in the proposed architecture. Because the MHA module operates on the spatially aggregated feature maps produced by SAP, a DenseNet201+MHA variant without SAP cannot be constructed without altering the data flow of the network. The reported ablation therefore isolates the incremental contribution of SAP (DenseNet201 vs. DenseNet201 + SAP) and of MHA given SAP (DenseNet201 + SAP vs. DenseNet201 + SAP + MHA). A design in which the two modules are fully independent is left for future work. The confusion matrices for each fold are presented in Figure 7. The DenseNet201_SAP_MHA model consistently shows high performance across all folds, with accuracy values generally exceeding 95%. For instance, in Fold 1, the accuracy for classifying “Bad” and “Good” target classes is 96.5% and 94.2%, respectively. This trend is maintained across all five folds, with minimal variation. The DenseNet201_SAP model also demonstrates strong performance, slightly lower than DenseNet201_SAP_MHA in some folds. For example, in Fold 1, it achieves 95.6% accuracy for the “Bad” class and 93.3% for the “Good” class. The performance remains relatively stable across all folds, with minor fluctuations. While the base model, DenseNet201, shows the lowest performance among the three models. Across all folds, DenseNet201_SAP_MHA consistently outperforms the other models, particularly in maintaining high classification accuracy for both “Bad” and “Good” target classes. The incorporation of SAP and MHA results in consistently higher performance across the five folds. Furthermore, DenseNet201_SAP shows an improvement over the base DenseNet201 model, suggesting that the addition of SAP contributes positively to the model’s performance. However, it is evident that combining both SAP and MHA (as in DenseNet201_SAP_MHA) yields the best results. The slight variations in accuracy across folds for all models highlight the natural variability in cross-validation. However, the consistent performance of DenseNet201_SAP_MHA indicates its stability across the cross-validation folds.

**Figure 8:**
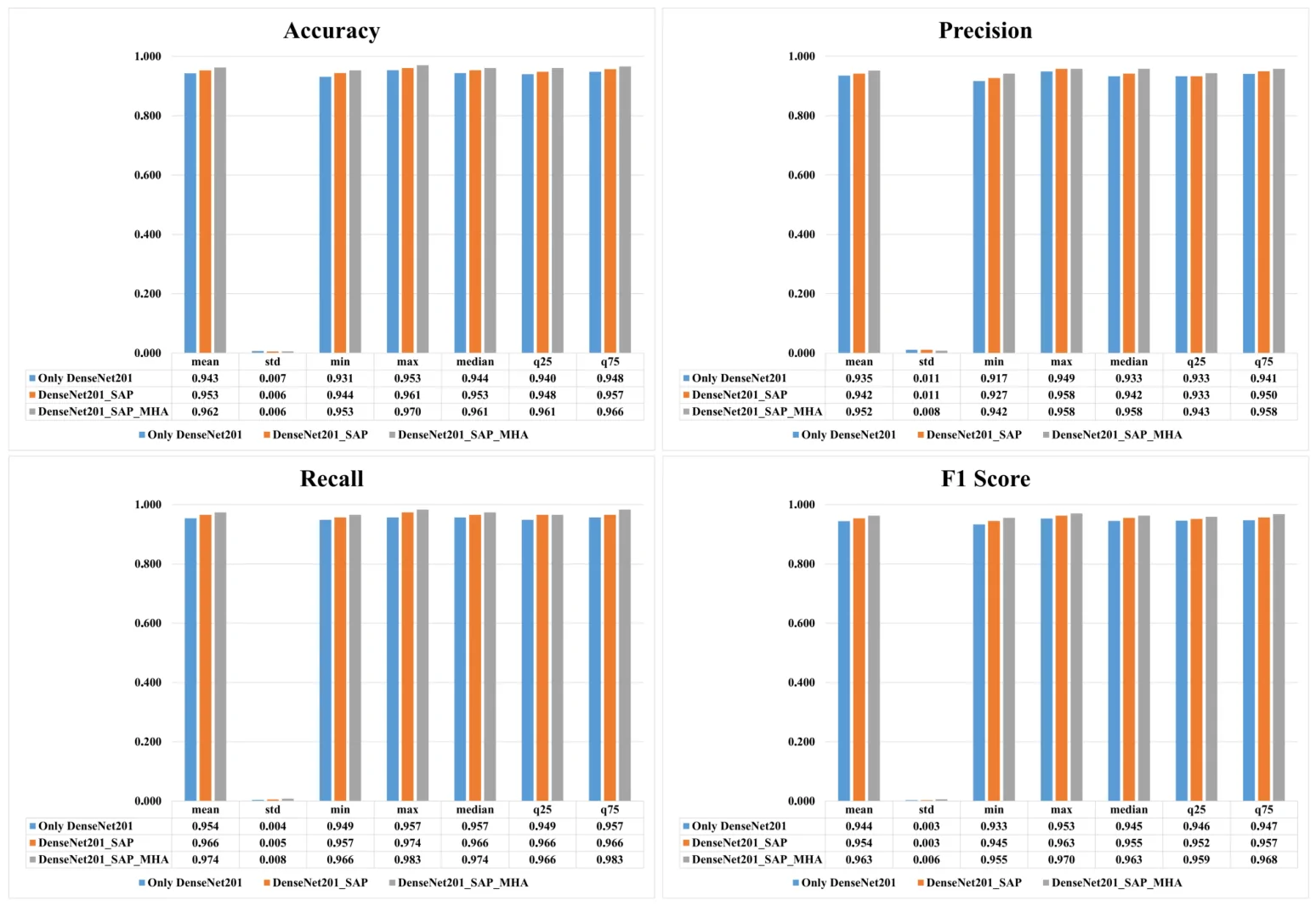
Comprehensive statistical summary of classification performance metrics from 5-fold cross-validation for DenseNet201, DenseNet201_SAP, and DenseNet201_SAP_MHA models.

Figure 8 summarizes the performance evaluation of the three structures through 5-fold cross-validation. This rigorous evaluation provides insights into their effectiveness in detecting bobbles, considering metrics such as accuracy, precision, recall, and F1-score. The results reveal notable distinctions in accuracy across the models. “Only DenseNet201” exhibits the lowest mean accuracy (0.943) among the evaluated models. In contrast, both “DenseNet201_SAP” and “DenseNet201_SAP_MHA” demonstrate improved performance metrics. Particularly, “DenseNet201_SAP_MHA” achieves the highest mean accuracy (0.962), suggesting that the integration of selective attention processing and multi-head attention mechanisms enhances the model’s predictive capabilities. Precision and recall metrics further emphasize the benefits of architectural enhancements. “DenseNet201_SAP_MHA” consistently achieves superior precision and recall compared to both “Only DenseNet201” and “DenseNet201_SAP”. These results indicate improved model sensitivity and specificity in detecting and classifying bubbles in tires. The F1-score, harmonizing precision and recall, underscores the comprehensive performance assessment across all metrics. “DenseNet201_SAP_MHA” emerges as the top performer with the highest F1-score across mean, standard deviation, minimum, maximum, median, q25, and q75 metrics. This outcome validates the effectiveness of incorporating selective attention processing and multi-head attention mechanisms in achieving a balanced trade-off between precision and recall, which is essential for robust tire bubble recognition systems.

Since the experiments were carried out on a class-balanced subset, the expected performance under the original class distribution (581 defective and 811 non-defective images) was also estimated. Sensitivity and specificity do not depend on the class proportions; computed from the pooled cross-validation results, they are 0.974 and 0.952 for DenseNet201_SAP_MHA, respectively. Reweighting these values to the original distribution gives an estimated accuracy of 0.961, a precision of 0.935 and an F1-score of 0.954 for the proposed model, compared with estimated accuracies of 0.951 for DenseNet201_SAP and 0.942 for DenseNet201; thus, the ranking of the three models is unchanged. Because the defect rate on the production line is lower than in either distribution, sensitivity and specificity are the most informative metrics for the practical use of the system.

**Figure 9:**
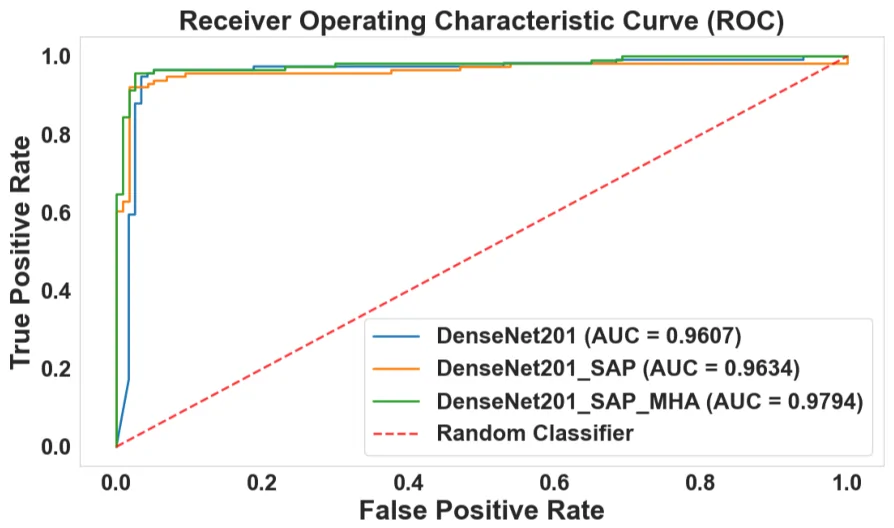
ROC curves for DenseNet201, DenseNet201_SAP, and DenseNet201_SAP_MHA models.

The performance of the three models in differentiating between bubble (Bad) and no bubble (Good) images was also evaluated using ROC curves AUC. The ROC curve plots the TPR on the y-axis against the False Positive Rate (FPR) on the x-axis. TPR, also known as sensitivity, represents the proportion of true bubble cases correctly classified by the model. Conversely, FPR, or 1-specificity, represents the proportion of non-bubble cases incorrectly classified as a bubble. A perfect classifier would achieve a TPR of 1 with an FPR of 0. The ROC curve for a perfect classifier would therefore lie along the upper left corner of the plot. Conversely, a random classifier would perform no better than chance, resulting in a ROC curve that follows a diagonal line from the bottom left to the top right corner. The ROC curves for the three structures are presented in Figure 9. As evident from the figure, the ROC curve for DenseNet201_SAP_MHA consistently lies above the curves for DenseNet201 and DenseNet201_SAP across all FPR values. This indicates that DenseNet201_SAP_MHA exhibits a superior ability to distinguish between bubble and non-bubble images compared to the other two models. This observation is further confirmed by the corresponding AUC values: DenseNet201_SAP_MHA achieves the highest AUC of 0.9794, followed by DenseNet201_SAP (0.9634) and DenseNet201 (0.9607).

For further investigation, we have included a Taylor diagram in our experiments. A Taylor diagram is a graphical representation used to evaluate the performance of different models by comparing their statistical properties with observed data. The Taylor diagram simultaneously displays three key statistics:

1. Correlation Coefficient: Measures the linear correlation between the model predictions and observed data. A correlation coefficient closer to 1 indicates a stronger agreement.
2. Standard Deviation: Reflects the variability of the model predictions. Ideally, the standard deviation of the model should match that of the observed data.
3. Root Mean Square Error (RMSE): Combines both the standard deviation and correlation to provide an overall measure of model performance.

**Figure 10:**
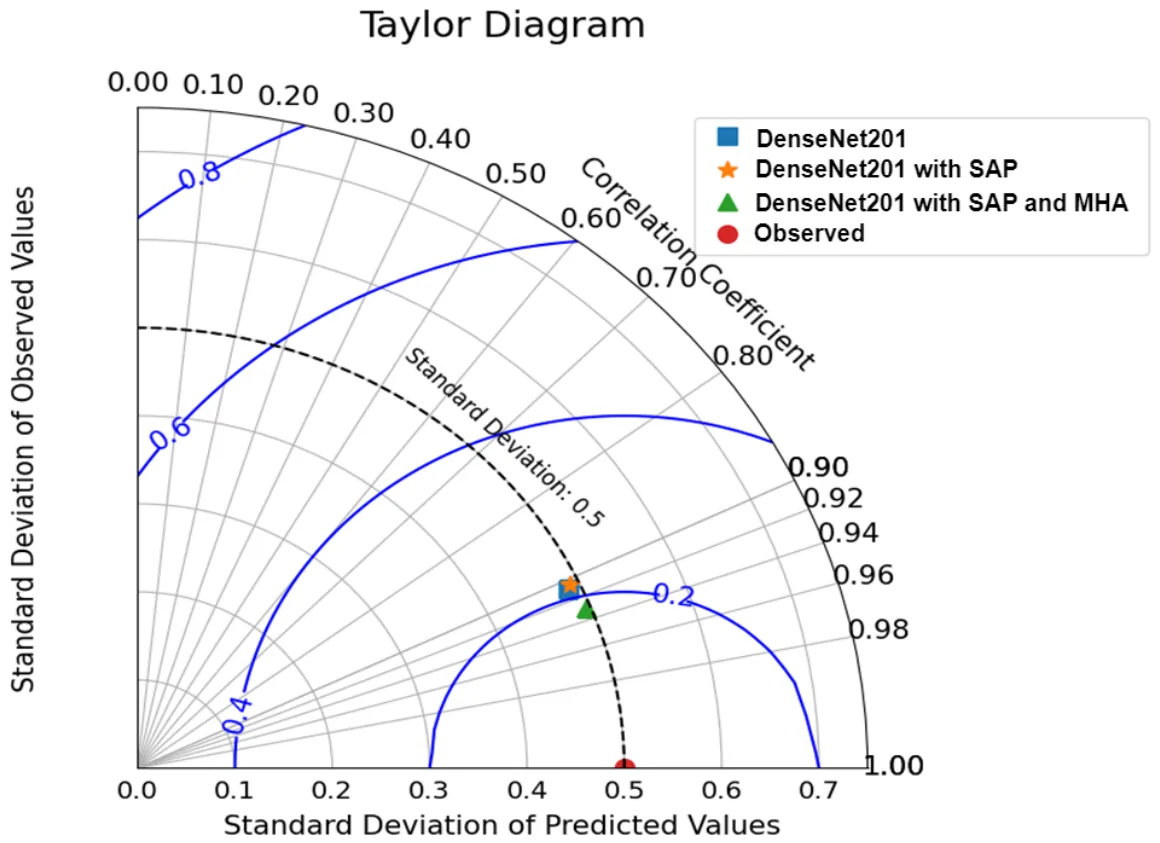
Taylor Diagram comparing the performance of DenseNet201, DenseNet201 with SAP, and DenseNet201 with SAP and MHA against observed data.

The Taylor diagrams offer a unified representation of multiple statistical measures, making it easier to compare the performance of different models. By visualizing multiple metrics on a single plot, Taylor diagrams reduce the complexity and time required for model assessment. They also provide a clear and concise way to identify which models perform best in terms of both accuracy and variability. Figure 10 presents the Taylor diagram comparing the performance of three models—DenseNet201, DenseNet201 with SAP, and DenseNet201 with SAP and MHA—against observed data. The key findings from the diagram are summarized as follows. The observed data has a standard deviation of approximately 0.5. Both DenseNet201 and DenseNet201 with SAP and MHA exhibit standard deviations close to the observed value, indicating a similar level of variability. DenseNet201 and DenseNet201 with SAP show a lower standard deviation, suggesting less variability in their predictions. DenseNet201 and DenseNet201 with SAP achieve a high correlation coefficient of around 0.92, indicating strong agreement with the observed data. DenseNet201 with SAP and MHA demonstrates the highest correlation, close to 0.94, signifying the best agreement with the observed data among the models. The blue RMSE contours help in visualizing the error magnitude between model predictions and observed data. The DenseNet201 with SAP and MHA emerges as the top-performing model, exhibiting the lowest error value. DenseNet201 with SAP and MHA emerges as the top-performing model, exhibiting the highest correlation with the observed data and a standard deviation close to the observed value. DenseNet201 and DenseNet201 with SAP also perform well, but with a slightly lower correlation.

## 5. Statistical Analysis

In this section, different statistical tests are used to examine the significant differences between the three structures statistically, which is an important step in analyzing the uncertainty of the models’ results [43]. To start with, the normality of the dataset was assessed using the Shapiro–Wilk test. This test evaluates the null hypothesis that the data was drawn from a normal distribution. The results for each model are presented in Table 1.

For the DenseNet201 model, the Shapiro–Wilk test yielded a test statistic of 0.9631 with a *p*-value of 0.8295. Since the *p*-value is significantly greater than 0.05, we fail to reject the null hypothesis, indicating that the data for this model follows a normal distribution. The DenseNet201_SAP model had a test statistic of 0.9000 and a *p*-value of 0.4096. Similar to the DenseNet201 model, the *p*-value for DenseNet201_SAP is well above the 0.05 threshold, suggesting that the data for this model is also normally distributed. In the case of the DenseNet201_SAP_MHA model, the Shapiro–Wilk test produced a test statistic of 0.7868 with a *p*-value of 0.0629. This *p*-value is slightly above the 0.05 threshold, indicating marginal non-normality. Although the *p*-value is close to the threshold, it is not low enough to reject the null hypothesis conclusively. Therefore, we can reasonably assume normality, but with some caution. Based on the Shapiro–Wilk test results, we can conclude that the data for the DenseNet201 and DenseNet201_SAP models are normally distributed. The DenseNet201_SAP_MHA model, while close to the threshold for non-normality, does not provide strong evidence against the assumption of normality. Therefore, parametric tests such as the paired *t*-test may be appropriate for these datasets, especially for the DenseNet201 and DenseNet201_SAP models. For the DenseNet201_SAP_MHA model, it may be prudent to consider non-parametric alternatives due to its borderline *p*-value for normality.

**Figure 11:**
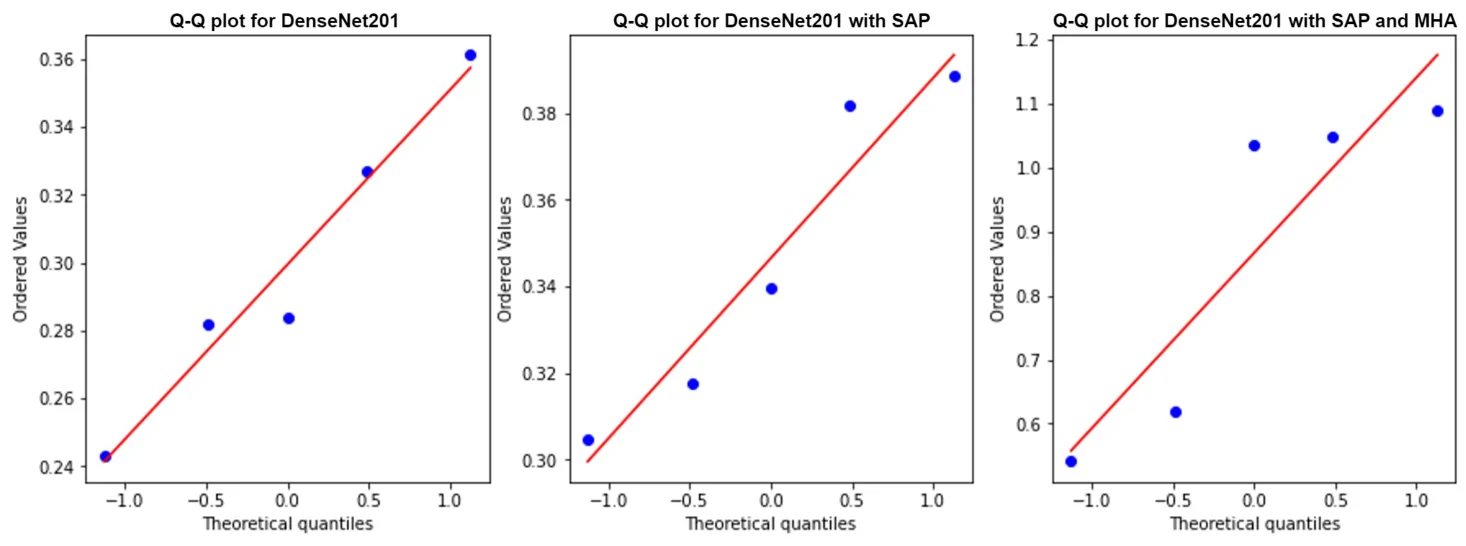
Q-Q plots for DenseNet201, DenseNet201_SAP, and DenseNet201_SAP_MHA.

To further check the normality of the models’ behavior, the Q-Q (quantile-quantile) plots were plotted for the DenseNet201, DenseNet201_SAP, and DenseNet201_SAP_MHA as shown in Figure 11.

The points for the three models lie close to the reference line, with minor deviations. This suggests that the three models’ results are approximately normally distributed. The slight deviations are within acceptable limits, indicating that the normality assumption is reasonable. To sum up, the Q-Q plots support the results of the Shapiro–Wilk test and add a visual confirmation of the normality test results, providing a comprehensive understanding of the data distribution for each model. Leveraging the normality of the data, we employed a one-way ANOVA to assess mean differences between the three models: DenseNet201, DenseNet201_SAP, and DenseNet201_SAP_MHA. For a more granular analysis, post-hoc *t*-tests were conducted to pinpoint specific pairwise variations.

The ANOVA test evaluates the null hypothesis that all group means are equal against the alternative hypothesis that at least one group mean is different. The results of the ANOVA test are as follows: the Test Statistic (F-value) is 20.388 and the *p*-value is 0.00014. The test statistic, or F-value, is 20.388, which is relatively high. This suggests that there is a substantial amount of variability between the group means compared to the variability within the groups. The *p*-value is 0.00014, which is significantly lower than the conventional threshold of 0.05. This indicates that we can reject the null hypothesis with a high level of confidence. In other words, there is strong evidence to conclude that there are significant differences between the means of the three models. Paired *t*-tests were conducted to compare the performance of the three models: DenseNet201, DenseNet201_SAP, and DenseNet201_SAP_MHA. The results of the *t*-tests are presented in Table 2.

The paired *t*-test evaluates whether the mean difference between paired observations is significantly different from zero. The results are as follows:

- DenseNet201 vs. DenseNet201_SAP: The t-statistic is −3.155512, and the *p*-value is 0.034332. Since the *p*-value is less than 0.05, we reject the null hypothesis and conclude that there is a significant difference between the performance of DenseNet201 and DenseNet201_SAP.
- DenseNet201 vs. DenseNet201_SAP_MHA: The t-statistic is −4.966281, and the *p*-value is 0.007672. With a *p*-value significantly less than 0.05, we reject the null hypothesis, indicating a significant difference in performance between DenseNet201 and DenseNet201_SAP_MHA.
- DenseNet201_SAP vs. DenseNet201_SAP_MHA: The t-statistic is −4.329718, and the *p*-value is 0.012352. This *p*-value is also less than 0.05, leading us to reject the null hypothesis and conclude that there is a significant difference between the performance of DenseNet201_SAP and DenseNet201_SAP_MHA.

The paired *t*-test results indicate significant differences in performance among the three models. Specifically, each pairwise comparison shows a statistically significant difference, suggesting that the models have distinct performance characteristics. These results reinforce the findings from the ANOVA test, providing further evidence that at least one model performs differently from the others. Given the small number of folds (*n* = 5), the statistical tests reported here should be interpreted as indicative rather than definitive. The strongest evidence for the contribution of the attention modules is the consistent direction and magnitude of the improvement across all five folds, rather than the *p*-values alone.

### 5.1. Insights into Model Explainability

Model explainability is crucial for understanding how deep learning models make predictions, particularly in safety-critical applications such as tire defect detection. In this study, we utilize two powerful model interpretability techniques: Grad-CAM and SHAP. These methods highlight which regions and features of the input images have the most influence on the models’ classification decisions [44].

**Figure 12:**
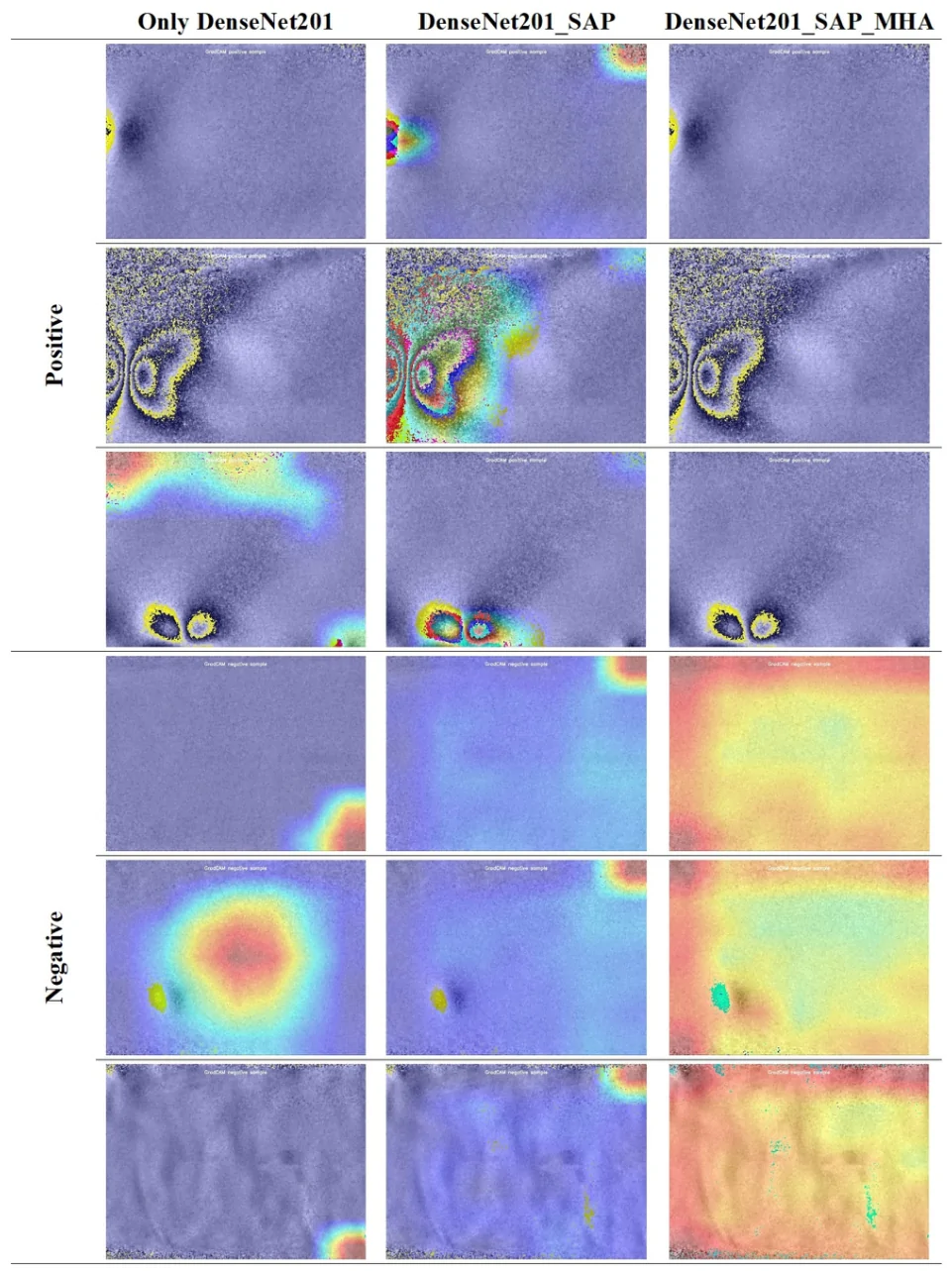
Grad-CAM visualizations for DenseNet201, DenseNet201_SAP, and DenseNet201_ SAP_MHA, showcasing the regions of input images that most strongly influence the classification decisions.

The Grad-CAM visualizations in Figure 12 exhibit how well each of the models—DenseNet201, DenseNet201_SAP, and DenseNet201_SAP_MHA—focus on crucial regions for tire bubble detection. In the case of DenseNet201, the model fails to focus solely on the bubble, instead expanding its attention to irrelevant areas, resulting in a lower performance. DenseNet201_SAP refines this focus, notably improving attention to bubble regions. DenseNet201_SAP_MHA enhances this capability. DenseNet201_SAP_MHA’s multi-scale attention locates bubbles with high precision, making it the best-performing model for bubble detection. The models behave differently in negative class images lacking bubbles. DenseNet201 and DenseNet201_SAP might focus on irrelevant regions, potentially misidentifying artifacts or noise as defects. For negative images, the Grad-CAM maps of DenseNet201_SAP_MHA do not show a localized salient region; the activation is spread over the image, which is consistent with the absence of a bubble-specific cue.

**Figure 13:**
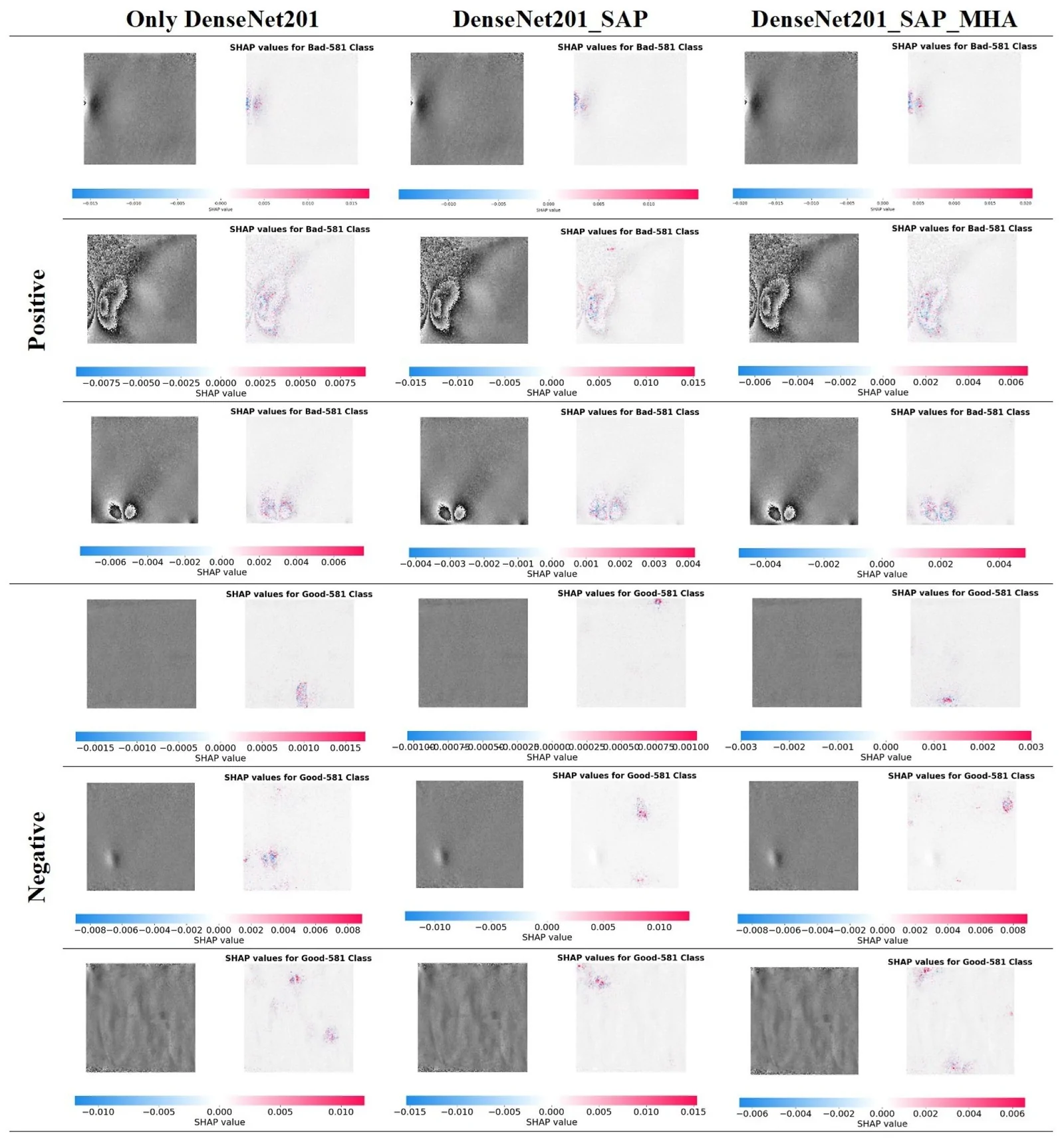
Visualization of SHAP values for model interpretability of DenseNet201, DenseNet201_ SAP, and DenseNet201_SAP_MHA, highlighting the contribution of each feature to the classification predictions.

The SHAP value visualizations in Figure 13 offer an alternative perspective on model interpretability by quantifying the importance of each feature to classification decisions. In the SHAP visualizations, each subfigure emphasizes the significance of particular factors in determining whether an image is positive (bubble present) or negative (bubble absent). The DenseNet201 model, like the Grad-CAM results, exhibits a less targeted interpretation, with the contribution from several irrelevant features influencing the prediction. DenseNet201_SAP improves on this by limiting the main characteristics relevant to bubble detection. However, some features that do not strongly correlate with the presence of bubbles maintain their influence. In contrast, DenseNet201_SAP_MHA delivers the most refined results, explicitly identifying and emphasizing the factors that significantly contribute to the model’s accurate predictions. The SHAP values demonstrate that DenseNet201_SAP_MHA is capable of not only detecting minor variations in image texture but also offering the highest importance to the most valuable features, delivering clear and explainable predictions. This detailed feature attribution makes DenseNet201_SAP_MHA the most interpretable and reliable model among the three. Together, these visualizations offer valuable insights into how attention mechanisms and multi-scale processing improve both the accuracy and interpretability of the model. By focusing on relevant regions and features, DenseNet201_SAP_MHA demonstrates superior performance in detecting tire bubbles, ensuring that critical areas are correctly identified while minimizing distractions from irrelevant data.

### 5.2. Failure Analysis

**Figure 14:**
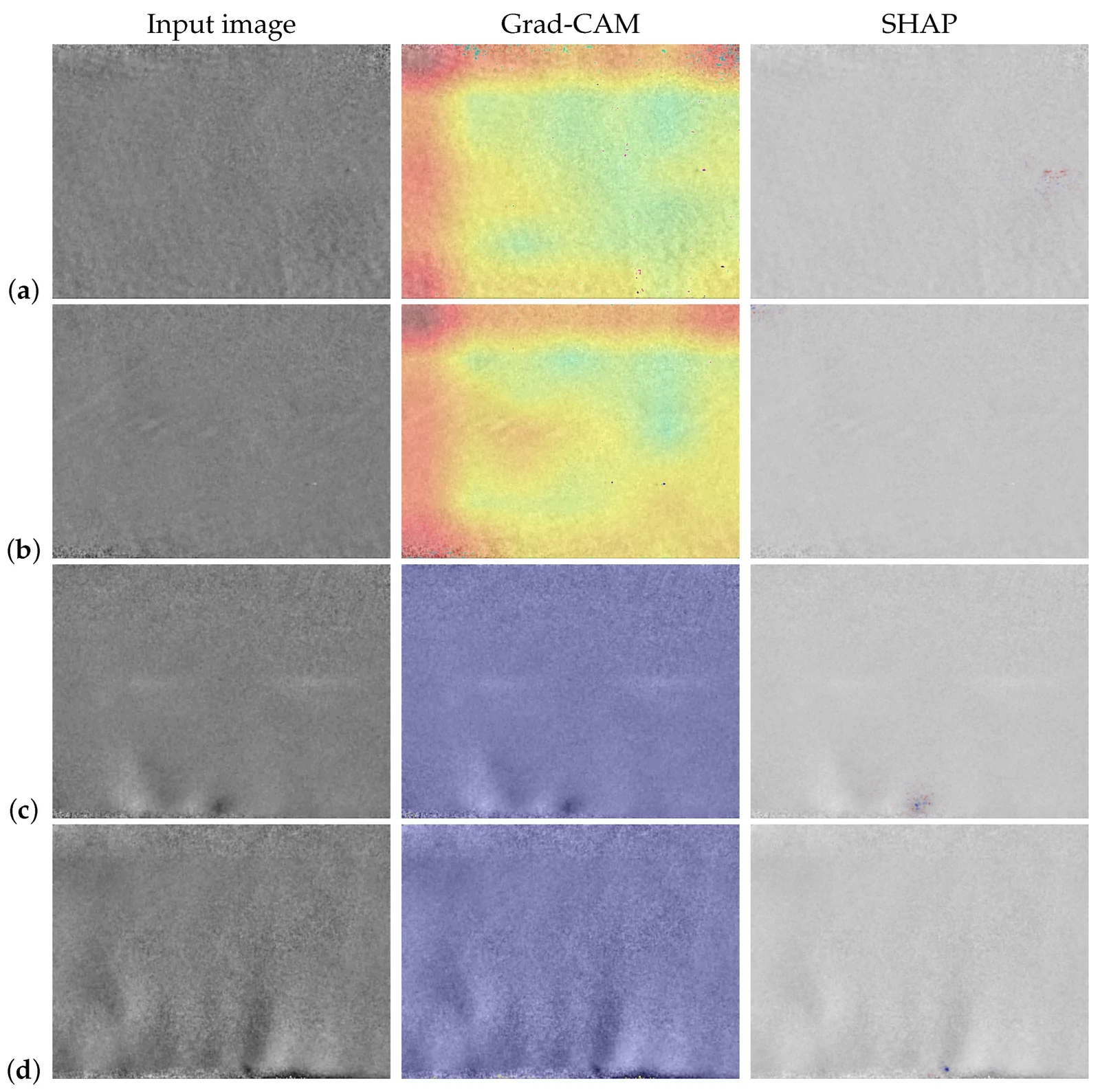
Grad-CAM and SHAP explanations of misclassified images for DenseNet201_SAP_MHA. (**a**,**b**) False negatives: images containing a bubble, predicted as non-defective (predicted probability of the non-defective class: 1.00 and 1.00). (**c**,**d**) False positives: non-defective images predicted as defective (predicted probability of the non-defective class: 0.18 and 0.00). Grad-CAM maps are normalized per image; in the SHAP maps, red and blue indicate positive and negative contributions to the non-defective class, respectively.

To examine whether the explanations are also informative for incorrect decisions, Grad-CAM and SHAP explanations were generated for the misclassified images of the proposed model, including both false negatives (images containing a bubble predicted as non-defective) and false positives (non-defective images predicted as defective). Two representative examples of each type are shown in Figure 14.

In the first false negative (Figure 14a), the bubble appears as a very small local disturbance on the right side of the image. SHAP concentrates its attribution precisely on this region, showing that the model does respond to the defect; however, the signature is too small to change the decision. In the second false negative (Figure 14b), the bubble is similarly small, but neither method highlights it; SHAP attributes the decision mainly to the speckle noise in the upper-left corner. In both false negatives, the Grad-CAM maps are broad and dominated by the image borders, without a localized response at the bubble.

In the first false positive (Figure 14c), the image contains a dark, round spot at the lower border surrounded by a local deformation pattern that closely resembles a bubble signature, and SHAP concentrates its attribution on this spot. In the second false positive (Figure 14d), the image contains periodic vertical stripes caused by the tread pattern and a dark spot at the lower border, on which SHAP again concentrates. In both false positives, Grad-CAM shows low activation over almost the entire image.

These cases indicate that the remaining errors are mainly associated with very small bubble signatures, which produce only weak local responses, and with non-defect structures that resemble bubble signatures, such as dark spots and local deformation patterns near the image borders or periodic tread-pattern structures. In these failure cases, SHAP localized the relevant structures more precisely than Grad-CAM, whose maps were broad and less specific, which suggests that the two methods are complementary. The explanation maps make the source of each error visible to the operator: when the attribution is concentrated on a border region, a noisy corner or a tread-pattern structure, the prediction can be checked more closely, which supports the human-in-the-loop use of the system on the production line.

### 5.3. Comparison with Literature

This section places the proposed method in the context of existing research on bubble defect detection in tire shearography images. Since the classification of tire bubbles in shearography images is a relatively recent field, only a limited number of studies are available. Table 3 summarizes these studies in terms of publication year, task, dataset size, model, explainability and reported accuracy.

Except for our previous study [20], these studies use different datasets, and several of them address object detection rather than image classification, with different evaluation protocols and metrics. Therefore, the reported accuracies are not directly comparable. A comparison on a common benchmark is not possible, since tire shearography images are acquired on industrial inspection lines and are subject to the confidentiality requirements of the manufacturers; to the best of our knowledge, none of the datasets used in these studies is publicly available, and the dataset used in this study can likewise be shared only on request because of the confidentiality agreement with the manufacturer.

The advantages of the proposed method can nevertheless be assessed on three grounds that do not depend on cross-dataset accuracies. First, on the same dataset, our previous study [20] compared six transfer learning backbones (VGG16, VGG19, ResNet50, Xception, MobileNetV2 and DenseNet201) and found DenseNet201 to perform best. In the present study, the proposed model outperformed DenseNet201 under identical data, folds and training settings, with consistent improvements in all five folds; this constitutes a controlled comparison against the best previously reported method on this dataset. Second, the proposed framework has properties that can be compared independently of the dataset. It explains its decisions through Grad-CAM and SHAP, which none of the previous studies provide; this is important in safety-critical inspection, where incorrect predictions may have substantial consequences and the basis of each decision must be verifiable. It also performs image-level classification, which directly matches the accept/reject decision of the inspection process and does not require the bounding-box annotations needed by the object detection approaches [8,30], and its performance is assessed through cross-validation with statistical testing. Third, its computational cost has been measured, with a mean inference time of 76.01 ms per image including preprocessing, and the system has been deployed on the production line.

### 5.4. Computational Cost and Explainability Overhead

The practical deployment of an inspection model depends on its size, inference latency, and the overhead introduced by explainability methods. Table 4 reports these quantities for the three variants. All measurements were obtained on the same workstation (NVIDIA RTX 4060 Laptop GPU with 8 GB VRAM; Grad-CAM and SHAP were executed on CPU). Inference time includes preprocessing (resizing to 224×224 and 1/255 rescaling) and the forward pass. Grad-CAM time comprises the backward pass and heatmap generation. SHAP time was measured with *GradientExplainer* using *nsamples* = 1 and a background set of two images. The proposed DenseNet201 + SAP + MHA model has 30,460,417 trainable parameters (30,767,297 total) and occupies 120.16 MB on disk. Its mean inference time is 76.01 ms (median 73.93 ms; range 64.42–136.56 ms), compared with 63.24 ms for plain DenseNet201 and 256.44 ms for DenseNet201 + SAP. The proposed model therefore introduces only a modest latency increase of approximately 12.8 ms over the plain backbone while avoiding the substantially higher inference cost of the SAP-only variant. This indicates that the attention modules do not compromise practical throughput; the proposed model can process roughly 13 images per second on the test workstation. For explainability, Grad-CAM requires 502.07 ms on average for the proposed model, versus 267.59 ms for DenseNet201 and 2038.46 ms for DenseNet201 + SAP. SHAP requires 1328.62 ms for the proposed model, 836.17 ms for DenseNet201, and 3342.98 ms for DenseNet201 + SAP. These costs are incurred only when an explanation is explicitly requested and therefore do not reduce the primary inspection throughput. The DenseNet201 + SAP variant is considerably slower for both inference and explainability.

## 6. Limitations

This study has several limitations. First, cross-validation was performed at the image level, so images of the same tire may appear in both training and test folds, which may make the estimates optimistic. Second, the model was not evaluated on an independent set of new tires and unseen tread patterns. Third, the explanations were assessed qualitatively; pixel-level expert annotations were not available for a quantitative evaluation, and masking-based faithfulness and stability analyses were not performed. Fourth, acquisitions affected by temperature or vibration were excluded and are handled by the operator in practice; their automatic recognition is left for future work. Fifth, the models were trained on a class-balanced subset, and performance under the original distribution was only estimated. Finally, a comparison on a common benchmark was not possible, since tire shearography datasets are not publicly available.

## 7. Conclusions

This work integrates DenseNet201 with advanced attention mechanisms, namely the SAP and MHA modules, to offer an innovative approach for bubble defect detection in tire shearography images. This research shows the significant impact of these improvements on model performance through rigorous experimentation and comparison with model modifications, leading to higher classification accuracy and more consistent performance across the cross-validation folds. The suggested DenseNet201_SAP_MHA model continuously beat DenseNet201 and DenseNet201_SAP on a number of performance measures, such as F1-score, accuracy, precision, and recall. In a 5-fold cross-validation, the model’s mean accuracy of 96.2% demonstrated its consistent performance in identifying bubbles. This result was also reflected in the ROC curve analysis and AUC values; with an AUC of 0.9794, it outperformed both DenseNet201_SAP and DenseNet201. In addition, the proposed model is computationally efficient, with a mean inference time of 76.01 ms per image, which supports real-time deployment on the production line.

The decision-making process of the model achieved a critical degree of interpretability and transparency with the addition of XAI tools like Grad-CAM and SHAP values. These techniques gave users a clearer picture of the model’s focus areas during bubble detection and a better grasp of the reasoning behind the model’s predictions. For safety-critical applications, like tire inspection, where reliability and system trust are crucial, this degree of explainability is necessary. The efficacy of the suggested approach was further supported by statistical analysis, which supported the performance differences between the model variants. These results support the study’s design decisions and highlight how crucial it is for industrial defect detection systems to incorporate attention mechanisms. Finally, this work offers a more accurate, interpretable, and high-performing AI system in critical inspection settings where precision and safety are paramount such as bubble detection of tires. Future research should examine how to apply this strategy to additional NDT problems and optimize XAI and attention mechanisms further to enhance flaw detection in various domains.

## Figures and Tables

**Table 1:** Shapiro–Wilk Test Results.

Model	Statistics	*p*-Value
DenseNet201	0.9631	0.8295
DenseNet201_SAP	0.9000	0.4096
DenseNet201_SAP_MHA	0.7868	0.0629

**Table 2:** Paired T-Test Results.

Comparison	t-Statistic	*p*-Value
DenseNet201 vs. DenseNet201_SAP	−3.155512	0.034332
DenseNet201 vs. DenseNet201_SAP_MHA	−4.966281	0.007672
DenseNet201_SAP vs. DenseNet201_SAP_MHA	−4.329718	0.012352

**Table 3:** Comparison with Existing Literature Studies.

Author	Year	Technology	Dataset Size	Model Name	Explainability	Accuracy %
[8]	2020	Shearography	4076	Faster Region-based CNN	No	89.16
[30]	2022	Shearography	2450	Incremental YOLOv3 SPP	No	95.40
[30]	2022	Shearography	2450	Modified YOLOv3 SPP	No	94.70
[20]	2024	Shearography	1392	DenseNet201 (transfer learning)	No	95.71
[34]	2022	Shearography	1000	MLP with GLCM	No	85.90
This Study	2026	Shearography	1162	DenseNet201 SAP MHA	Yes	96.20

**Table 4:** Computational cost and explainability overhead of the three model variants.

	Model Size			Inference Time (ms)		
Model	Disk (MB)	Trainable Params	Total Params	Mean	Median	Min/Max
DenseNet201 + SAP + MHA	120.16	30,460,417	30,767,297	76.01	73.93	64.42/136.56
DenseNet201 + SAP	118.14	29,934,337	30,241,217	256.44	286.24	69.83/546.40
DenseNet201	88.93	22,784,513	23,017,409	63.24	61.96	53.66/88.58
	**Grad-CAM Time (ms)**			**SHAP Time (ms)**		
**Model**	**Mean**	**Median**	**Min / Max**	**Mean**	**Median**	**Min/Max**
DenseNet201 + SAP + MHA	502.07	484.16	435.04/646.31	1328.62	1324.20	1321.78/1344.31
DenseNet201 + SAP	2038.46	1935.58	1627.14/2732.28	3342.98	3334.54	3145.61/3557.23
DenseNet201	267.59	266.51	262.20/276.14	836.17	842.79	810.95/848.17

## Data Availability

The data presented in this study are available from the corresponding author on reasonable request (privacy).
